# Bayesian Uncertainty Quantification for A Fractional-Order Model of the Human Ear

**DOI:** 10.1007/s10162-026-01058-8

**Published:** 2026-06-08

**Authors:** Prakash KC, Maryam Naghibolhosseini, Mohsen Zayernouri

**Affiliations:** 1 Department of Mechanical Engineering, Michigan State University, 428 S. Shaw Lane, East Lansing, 48824 MI USA; 2 Department of Communicative Sciences and Disorders, Michigan State University, 1026 Red Cedar Rd., East Lansing, MI 48824 USA

**Keywords:** Human ear, Bayesian inference, MCMC, DPOAE, Fractional-order modeling, Transfer function, Outer-middle ear gain

## Abstract

We employ the Hamiltonian Monte Carlo (HMC) algorithm to estimate model parameters and quantify their uncertainties in a fractional-order lumped-element model of the human ear in a Bayesian inference framework. The model, originally developed by Naghibolhosseini and Long (2018), incorporates fractional-order elements to capture viscoelastic memory effects in ear tissues that otherwise cannot adequately be represented via conventional integer-order models. Using previously optimized model parameters to construct informative priors, we perform Bayesian parameter estimation via the No-U-Turn Sampler (NUTS) implementation. From the inferred posterior distributions, we compute the model’s outer-middle ear gain (OMEG) and validate predictions against experimental OMEG derived from DPOAE measurements. Additionally, we compare stapes velocity transfer functions and ear canal pressure gain with established experimental and computational literature. HMC sampling yields well-convergent posterior distributions for all parameters, centered near original optimized values with uncertainty quantified through credible intervals. The posterior predictive OMEG frequency response closely matches the experimental OMEG measurements. Interestingly, the Bayesian-derived parameter sets correctly exhibit stapes velocity resonances near 1 kHz and ear canal pressure gain peaks between 2.5 and 4 kHz, with amplification ranging from 4 to 12 dB, consistent with cadaveric experimental measurements and existing computational models. The model demonstrates a minimal intersubject variability while capturing realistic biological variations within experimentally reported ranges. The present Bayesian HMC simulation approach then provides a robust uncertainty quantification for fractional-order ear model parameter inference, maintaining physiologically consistent predictions across multiple validation datasets. Hence, the proposed framework enhances the model’s credibility by establishing a firm foundation for further developing probabilistic diagnostic tools for hearing assessment in the future.

## Introduction

The human ear is a complex biological system composed of viscoelastic materials with power-law characteristics, non-local interactions, and memory dependence. Therefore, accurate computational modeling of the human ear, incorporating such viscoelastic behaviors, is essential to understand the basic mechanics of hearing toward developing more objective hearing assessment tools and designing auditory implants with increased precision in the future. Traditional lumped-element models, often employing classic constitutive laws such as Voigt or Maxwell models, have been commonly used to simulate such complex biosystem [1–6]. However, these conventional integer-order lumped-element models face limitations in fully describing the memory-dependent viscoelastic behavior of biomaterials and cannot adequately account for the power-law responses experimentally observed in such materials [7–12].

Fractional calculus offers a more generalized mathematical modeling approach by extending ordinary differentiation and integration to their fractional-order counterparts [13–16]. This approach has gained considerable interest in multiple fields due to its ability to naturally incorporate power-law effects, non-local interactions, and memory dependence through the definition of fractional-order derivatives [17–20]. Fractional-order modeling has proven to be successful in describing the dynamics of viscoelastic materials in various biological applications, including human arteries [7, 21], respiratory system [22], brain matter [23, 24], blood flow [25], kidney and liver tissue [26, 27], bladder tissues [28], and red blood cells [29, 30]. A key advantage is that a fractional-order element can interpolate between pure Newtonian viscous and Hookian elastic elements by tuning a single parameter, thus exhibiting a continuous relaxation distribution, making them ideal for simulating stress–strain relationships in viscoelastic materials [31–33].

Based on these applications, Naghibolhosseini and Long (2018) [34] developed a fractional-order human ear model, which is better adapted to the physical nature of biomaterials and accounts for the power-law characteristics of viscoelastic biotissues. Their study proposed that dynamics of viscoelastic ligaments, muscles, membranes, and joints within the ear can be more effectively modeled by incorporating their complex effects and multi-scale properties using fractional-order lumped elements. Most importantly, their modeling approach distinguished itself by using experimental measurements from living humans for parameter fitting and validation, in contrast to many previous models such as Zwislocki’s model [35] and O’Connor and Puria’s model [36] that relied on cadaveric data, in which tissues can lose their original viscoelastic properties [37]. Naghibolhosseini and Long (2018) estimated the proposed model’s parameters by fitting the model estimates to the ear canal input impedance measurements using wideband reflectance, and the model was validated by comparing its round-trip outer-middle ear gain (OMEG) estimates with those obtained from an elaborate experimental design using distortion product otoacoustic emissions (DPOAEs) [38]. Their fractional model demonstrated better agreement with experimental data and significantly reduced normalized model errors [36] compared to previous integer-order lumped models.

Building on the framework established by Naghibolhosseini and Long (2018) [34], the present study addresses parameter uncertainty in the fractional-order human ear model through Bayesian inference using Hamiltonian Monte Carlo (HMC) sampling. While the original work successfully optimized model parameters to fit experimental data using a deterministic least-squares method, this approach provides only point estimates and does not capture the inherent uncertainties or variability in biological systems and measurements. The importance of accounting for such uncertainty is underscored by studies showing that middle ear sound transmission is highly sensitive to variations in material properties [39]. Posterior distributions are obtained using HMC, and model predictions are validated against experimental OMEG measurements from DPOAE data, as well as stapes velocity transfer functions and ear canal pressure gain reported in the literature. This probabilistic framework enhances model reliability and provides a robust foundation for future model-based hearing assessment tools.

This work is structured as follows: First, we explain the overall methodology, including the fractional-order model and Bayesian estimation using Hamiltonian Monte Carlo for the uncertainty analysis. Next, we present results of the posterior parameter distributions and model validation with experimental data and existing literature. Finally, we conclude the relevance of these findings for auditory modeling and potential clinical applications.

**Fig. 1:**
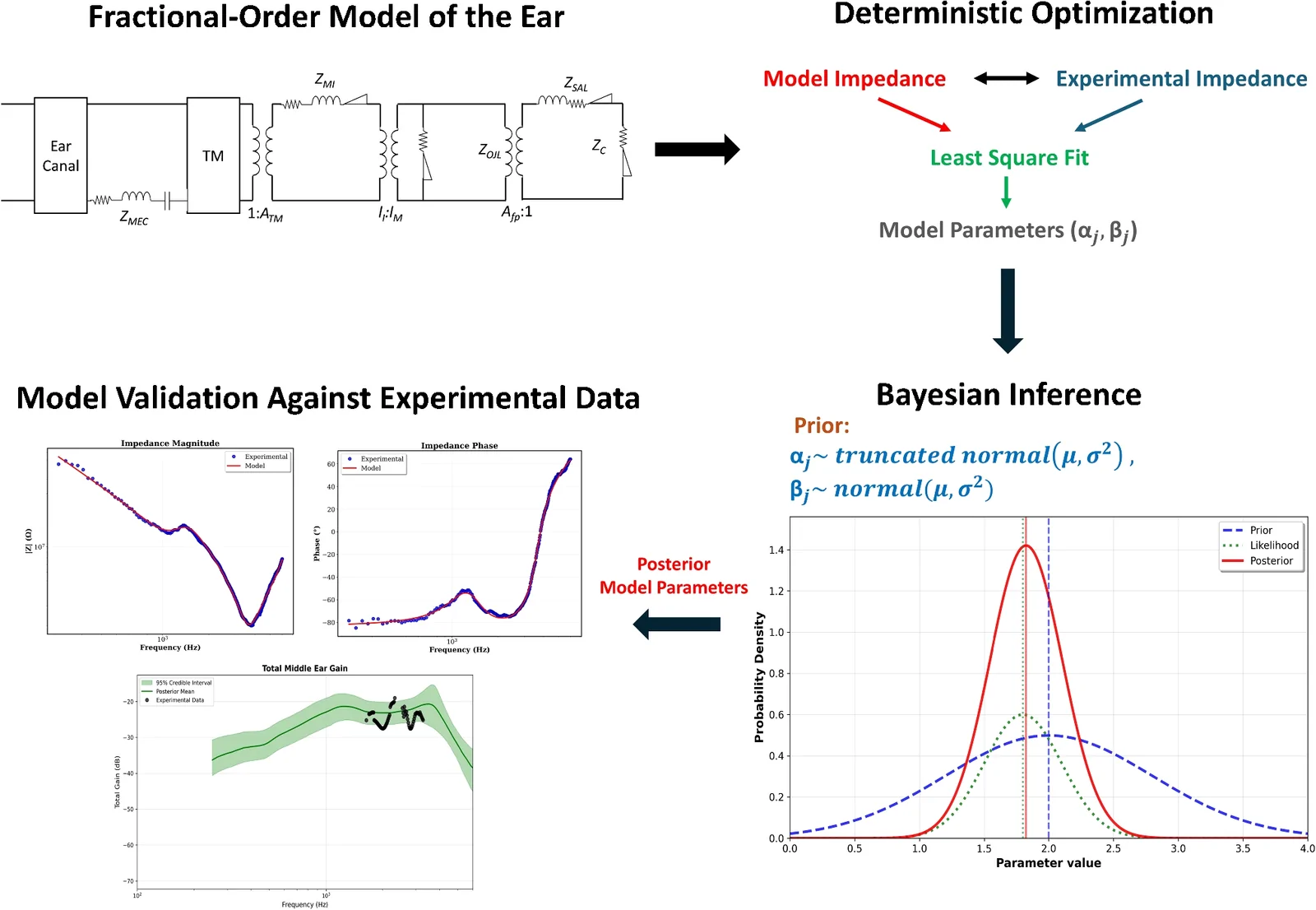
A schematic representation of the modeling framework illustrating: (i) the use of parameter point estimates obtained from deterministic least-squares optimization to construct informative priors for Bayesian inference, (ii) the inference of posterior parameter distributions via HMC sampling, and (iii) the comparison of posterior-based model predictions with experimental measurements for validation

## Methodology

We illustrate the modeling framework schematically in Fig. 1. As shown in the figure, the point estimates obtained from the deterministic least-squares optimization are used to construct informative prior distributions. These priors, modeled using truncated normal and normal distributions, form the basis of a Bayesian inference that quantifies parameter uncertainty through posterior distributions. The figure also captures how the posterior parameters are used to estimate data for comparison with experimental data towards the model validation. This approach not only captures the inherent variability in parameter estimates but also facilitates robust model validation by providing credible uncertainty bounds essential for reliable physiologic modeling and prediction. Each part of the modeling framework in Fig. 1 is explained comprehensively in what follows.

### Fractional-Order Lumped-Element Ear Model

Naghibolhosseini and Long (2018) [34] developed a comprehensive fractional-order lumped-element model of the human ear that addresses the limitations of conventional integer-order models in capturing the viscoelastic behaviors of biological tissues.

#### Model Architecture and Components

**Fig. 2:**
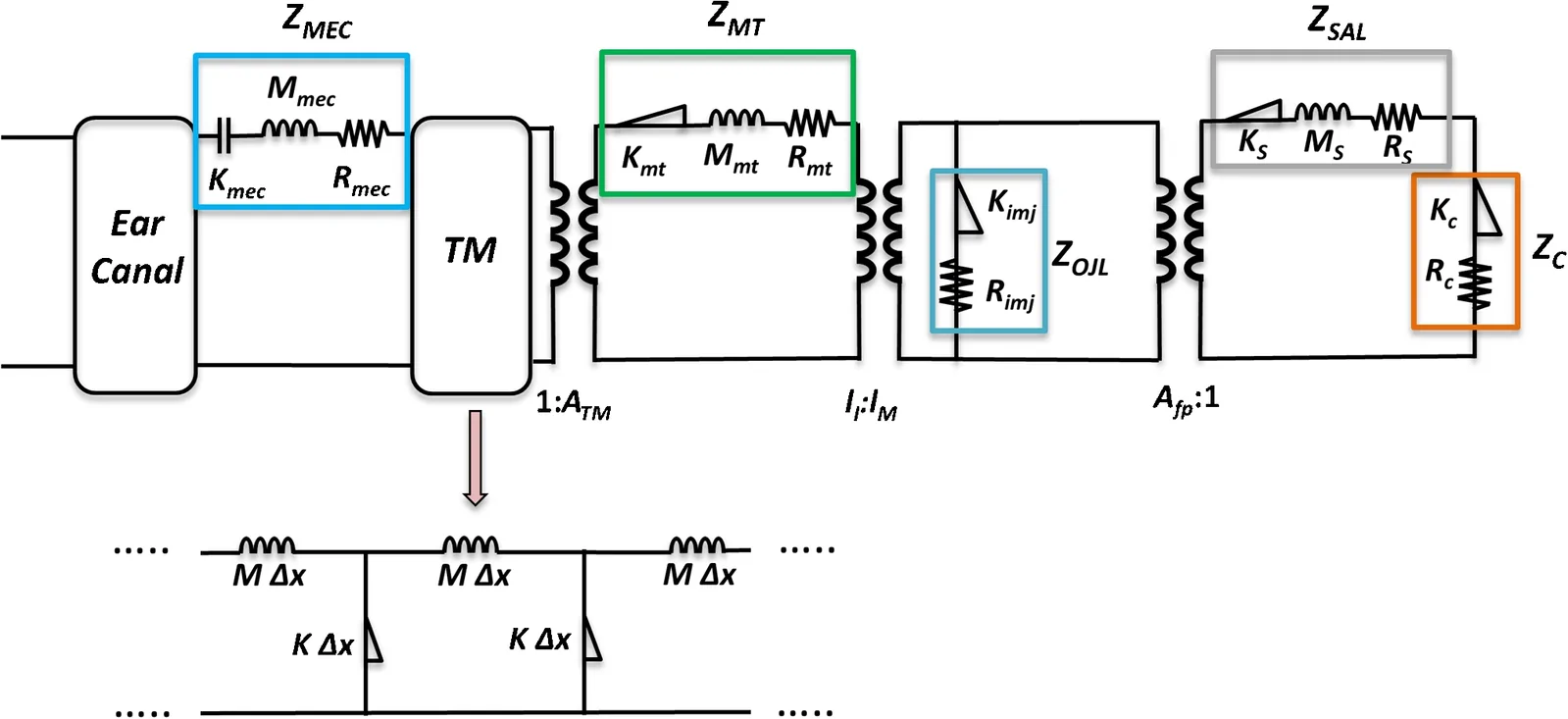
Fractional-order model of the human ear [34], where $Z_{MT}$ represents the impedance of the malleus and incus, $Z_{MEC}$ represents the impedance of the middle ear cavity, $Z_{OJL}$ represents the impedance of the ossicular joints, ligaments, and muscles, $Z_{SAL}$ represents the impedance of the stapes and annular ligaments, and $Z_c$ represents the impedance of the cochlea

The proposed model, illustrated in Fig. 2, is based on the established lumped model by O’Connor and Puria [36] but incorporates fractional-order elements and a fractional-order transmission line instead of classic integer-order components. The electrical elements of inductors, capacitors, and resistors are equivalent to the mechanical elements of masses, springs, and dashpots, respectively.

As shown in the figure, the ear canal is modeled as a lossless transmission line. Due to its inherent viscoelasticity, the tympanic membrane (TM) is uniquely represented by a fractional-order transmission line where fractional capacitors are implemented instead of conventional integer-order capacitors. The middle ear cavity (MEC), an air-filled space, is modeled as a series combination of an integer-order capacitor, a resistor, and an inductor $(Z_{MEC})$. Furthermore, the ossicular chain, comprising the malleus and incus bones, is represented as an inductor in series with a resistor and a fractional capacitor. These elements along with their parallel counterparts $(Z_{OJL})$ collectively account for the viscoelastic tissues attached to the malleus, incus, and stapes, such as ligaments, joints, and muscles. Electrical transformers account for changes in the pressure wave through the ear due to force reinforcement by the middle ear.

#### Governing Fractional-Order Differential Equations

The mathematical foundation of the model relies on fractional calculus definitions. The left Riemann–Liouville fractional integral of *f*(*t*) with order 0<α<1 is given by the following:

1 $$\begin{aligned} {}^{RL}_0I_t^\alpha f(t) = \frac{1}{\Gamma (\alpha )} \int _0^t \frac{f(s) \, ds}{(t-s)^{1-\alpha }}, \end{aligned}$$

in which Γ is the gamma function; the left-sided Caputo fractional derivative of order α is defined as follows:

2 $$\begin{aligned} ({}^C_0D_t^\alpha f)(t) = ({}^{RL}_0I_t^{1-\alpha } \frac{df}{dt})(t) = \frac{1}{\Gamma (1-\alpha )} \int _0^t \frac{f'(s) \, ds}{(t-s)^\alpha } \end{aligned}$$

For the fractional-order transmission line modeling the TM, the governing equations are derived by applying Kirchhoff’s law. When capacitors are considered as fractional-order elements, the conventional equation transforms into the following:

3 $$\begin{aligned} \frac{\partial ^2 e(x,t)}{\partial x^2} - LC \frac{\partial ^{\alpha +1} e}{\partial t^{\alpha +1}} = 0, \end{aligned}$$

where α is the fractional order of the derivative, *L* is the inductance, and *e* and *i* denote the electric potential and current.

In this formulation, the fractional capacitor is governed by $i = C\,\mathcal {D}_t^{\alpha } e$, or equivalently $i = C(j\omega )^{\alpha } e$ in the frequency domain. When α=1, this reduces to a classical capacitor and recovers the standard lossless wave equation; when α=0, the element becomes purely resistive, and the constitutive law is frequency-independent. Values 0<α<1 describe intermediate, frequency-dependent behavior characteristic of viscoelastic media such as the tympanic membrane. Under the impedance analogy adopted in this work, a capacitor is taken to be equivalent to a spring, since both elements store energy and exhibit an impedance proportional to 1/*jw* in the classical (integer-order) case. Consistently, a resistor corresponds to a dashpot, representing purely dissipative behavior. With this mapping, the fractional capacitor provides a direct analog of a fractional viscoelastic element. Specifically, α=1 corresponds to ideal capacitive behavior, which maps to a purely elastic spring; α=0 corresponds to resistive behavior, which maps to a purely viscous dashpot, and 0<α<1 captures intermediate viscoelastic behavior.

The input impedance along the fractional-order transmission line after taking a Laplace transform is defined as follows:

4 $$\begin{aligned} Z_{0\textrm{TM}}\, \frac{e^{T_{\textrm{TM}}\sqrt{{s^{\alpha +1}}}} + R\,e^{-T_{\textrm{TM}}\sqrt{{s^{\alpha +1}}}}}{e^{T_{\textrm{TM}}\sqrt{{s^{\alpha +1}}}} - R\,e^{-T_{\textrm{TM}}\sqrt{{s^{\alpha +1}}}} }, \end{aligned}$$

where $Z_{0TM}$ is the characteristic impedance, *R* is the reflection coefficient, and $T_{\textrm{TM}}$ is the wave propagation delay. The reflection coefficient *R* in this equation can be computed as follows:

5 $$\begin{aligned} R = \frac{Z_{out} - Z_{0TM}}{Z_{out} + Z_{0TM}}, \end{aligned}$$

where $Z_{out}$ is the impedance at the transmission line terminal.

Similarly, the input impedance at the ear canal is defined as follows:

6 $$\begin{aligned} Z_{in_{EC}} = Z_{0EC}\frac{e^{s(l_{ec}/C_0)}+ Re^{-{s(l_{ec}/C_0)}}}{e^{s(l_{ec}/C_0)} - Re^{-{s(l_{ec}/C_0)}}}, \end{aligned}$$

where $Z_{0EC}$ is the characteristic impedance of ear canal, $l_{ec}$ is the length of ear canal, and $C_0$ is the speed of sound.

#### Model Optimization and Validation

The development of the model involved a rigorous process of acquisition of experimental data, subsequent fitting of parameters (optimization), and a detailed validation of the model. Experimental data were collected non-invasively from seven normal-hearing adult participants in this study (four females, NDP1, NDP3, NDP5, and NDP12; and three males, NDP2, NDP6, and NDP11). Ear canal input impedance estimates, crucial for parameter fitting, were obtained through wideband reflectance measurements using a Hear ID Middle-Ear Power Analyzer MEPA3 (Mimosa Acoustics Inc., Champaign, IL). The input impedance is defined as the impedance seen from the probe location at the outer end of the ear canal, incorporating the combined contributions of the ear canal, tympanic membrane, and middle ear structures. This involved delivering a 60 dB SPL chirp stimulus and calibrating the system with four different cavities. The round-trip outer-middle-ear gain (OMEG) estimates, used for model evaluation, were derived from DPOAEs. DPOAEs were generated by presenting two or three external tones in the ear canal, with responses recorded via a custom Mac software, connected to an audio interface and a preamplifier (see [40] for more details). Furthermore, probe-microphone impedance, necessary for reverse transmission calculations, was determined through pressure measurements in five different lengths of a brass cavity. For model optimization, the 18 unknown parameters of the fractional-order model were determined by fitting the model to the measured impedance magnitude and phase estimates, specifically minimizing the sum of squared errors between the model’s output and the experimental reflectance data. For the detailed optimization algorithm, refer to [40]. Finally, the model validation (calibration) was performed by comparing the model’s OMEG estimates with the OMEG estimates derived from the DPOAE two- and three-tone paradigm measurements.

### Bayesian Analysis for the Impedance Model

While the deterministic optimization process described above provides point estimates for the model parameters, it does not quantify the uncertainty associated with these estimates. In reality, parameter uncertainty is inevitable and arises from multiple sources: measurement noise in the experimental data, inherent biological variability, and potential model structure limitations. To address this limitation, we use Bayesian inference to obtain full posterior probability distributions for the model parameters. The Bayesian inference uses prior knowledge about parameter values with the information contained in the experimental data to yield posterior distributions that quantify our updated knowledge about each parameter. This probabilistic framework not only provides point estimates but also credible intervals that characterize the range of possible parameter values given the data.

#### Prior Distribution Specification

We construct informative prior distributions based on the parameter values obtained from the deterministic optimization. For fractional-order exponents (i.e., the α parameters), we use truncated normal distributions to ensure that these parameters remain within physically meaningful bounds:

7 $$\begin{aligned} \alpha _j \sim \text {TruncatedNormal}(\mu _j, \sigma _j^2, \text {lower}=0.5, \text {upper}=1.0) \end{aligned}$$

The truncation bounds reflect physical constraints: values near 0.5 represent viscoelastic behavior with substantial memory effects, while values approaching 1.0 correspond to elastic (spring-like) behavior.

For all other parameters (stiffnesses, resistances, masses, characteristic impedances, and geometric parameters), we use normal prior distributions:

8 $$\begin{aligned} \beta _j \sim \text {Normal}(\mu _j, \sigma _j^2) \end{aligned}$$

The means $\mu _j$ are set to the optimized estimates obtained from the deterministic optimization, and the standard deviations $\sigma _j$ are chosen to be 10–20% of the absolute parameter values. We consider the optimized values as reasonable starting points and allow the data to shift these values within a reasonable range during the Bayesian optimizing process.

#### Likelihood Function

The likelihood function quantifies how probable the observed data are for any given set of parameter values. Since the impedance is a complex-valued quantity, we treat the real and imaginary components separately, assuming they have independent Gaussian measurement errors. This assumption is reasonable for the reflectance-based impedance measurements, where random noise in the time-domain reflectance signal propagates to both real and imaginary components of the frequency-domain impedance.

The likelihood function for the real and imaginary experimental impedance data $Z_{exp,re}$ and $Z_{exp,im}$ given model parameters $q_j$ and the noise’s real and imaginary standard deviations $\sigma _{re}$ and $\sigma _{im}$ is as follows:

9 $$\begin{aligned} \begin{aligned}&p(Z_{exp,re}, Z_{exp,im}|q_j, \sigma _{re}, \sigma _{im}) = \\&\qquad \qquad \qquad \quad \quad \prod _{i=1}^{N}\frac{1}{\sqrt{2\pi \sigma _{re}^2}} \exp \left[ -\frac{\left( Z_{exp,re}(\omega _i) - Z_{mod,re}(\omega _i;q_j)\right) ^2}{2\sigma _{re}^2}\right] \\&\qquad \qquad \qquad \qquad \qquad \qquad \qquad \times \frac{1}{\sqrt{2\pi \sigma _{im}^2}} \exp \left[ -\frac{\left( Z_{exp,im}(\omega _i) - Z_{mod,im}(\omega _i;q_j)\right) ^2}{2\sigma _{im}^2}\right] \end{aligned} \end{aligned}$$

for all *N* frequency points in the experimental dataset. In the above equation, $Z_{mod,re}$ and $Z_{mod,im}$ denote the model’s real and imaginary components of the impedance. The noise parameters $\sigma _{re}$ and $\sigma _{im}$ can either be estimated from replicate measurements or treated as hyperparameters with their own prior distributions and estimated jointly with the model parameters. In this study, the latter approach was implemented.

#### Posterior Distribution and Bayes’ Theorem

Bayes’ theorem provides the foundation for updating our prior beliefs about the parameters provided experimental evidence:

10 $$\begin{aligned} p(q_j|Z_{exp,re}, Z_{exp,im}) = \frac{p(Z_{exp,re}, Z_{exp,im}|q_j) p(q_j)}{\int p(Z_{exp,re}, Z_{exp,im}|q_j) p(q_j) dq_j}, \end{aligned}$$

where $p(q_j|Z_{exp,re}, Z_{exp,im})$ is the posterior distribution representing our updated knowledge about the parameters after observing the data. In the above equation, $p(Z_{exp,re}, Z_{exp,im}|q_j)$ is the likelihood function, $p(q_j)$ is the prior distribution, and the denominator is the marginal likelihood which serves as a normalization constant.

In practice, computing the evidence integral in the denominator is often difficult for complex models with many parameters. Fortunately, Markov chain Monte Carlo (MCMC) methods allow us to sample from the posterior distribution without explicitly computing this normalization constant.

#### Hamiltonian Monte Carlo Sampling

We use Hamiltonian Monte Carlo (HMC) as implemented in the PyMC probabilistic programming framework in Python [41, 42]. HMC is a gradient-based MCMC algorithm that is particularly efficient for exploring high-dimensional posterior distributions, especially when parameters are correlated. The key idea behind HMC is to treat the negative log-posterior as a potential energy function in a physical system and introduce auxiliary momentum variables. By simulating Hamiltonian dynamics-the same equations that govern the motion of particles in classical mechanics-the algorithm proposes new parameter values that can traverse the parameter space much more efficiently than simple random-walk proposals. Because Hamiltonian dynamics preserve volume in the phase space and are time-reversible, the algorithm maintains a detailed balance, ensuring that the samples converge to the true posterior distribution.

We specifically use the No-U-Turn Sampler (NUTS), an adaptive variant of HMC developed by Hoffman and Gelman in 2014 [43]. NUTS automatically tunes two critical algorithm parameters: the step size that controls how far each leap-frog integration step moves in the parameter space and the number of leap-frog steps that determines the length of each trajectory. NUTS uses a recursive algorithm to build a binary tree of candidate states along each trajectory. This adaptive approach eliminates the need for manual tuning while maintaining a high sampling efficiency.

Our MCMC configuration consists of 20 parallel chains and 5000 tuning steps to achieve an optimal performance. After tuning, each chain collects samples from the posterior distribution, yielding a total of 1,000,000 samples across all chains. This ensures reliable estimations of posterior means, standard deviations, and credible intervals even for complex, high-dimensional distributions. We compute convergence diagnostics including the effective sample size (ESS) for each parameter to account for the autocorrelation in the chains. A high ESS (typically > 400) indicates thorough exploration of the posterior distribution and verifies that no divergences have occurred. Divergences indicate potential problems with the Hamiltonian dynamics in regions of high posterior curvature.

**Fig. 3:**
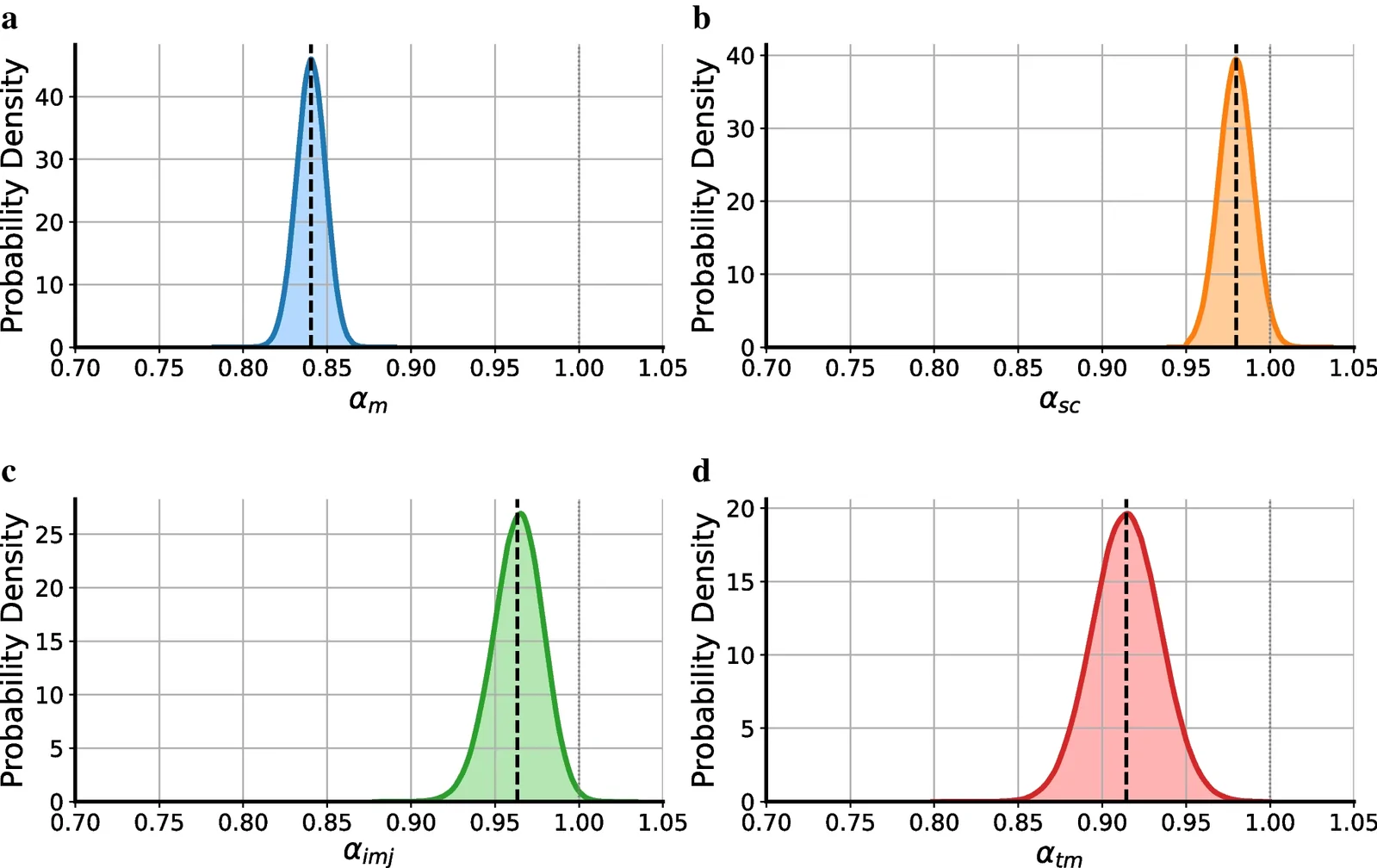
Posterior distributions for the fractional-order exponents (shown on the x-axes) of the NDP11 subject. (**a**) $\alpha _m$ governing the malleus-incus complex, (**b**) $\alpha _{sc}$ for the stapes-cochlea connection, (**c**) $\alpha _{imj}$ for the incudostapedial and incudomalleolar joints, and (**d**) $\alpha _{tm}$ for the tympanic membrane

Similar Bayesian approaches have been successfully applied to constitutive modeling of biological tissues in previous studies [44–46]. Of particular relevance, Miles et al. [47] implemented Bayesian inference to quantify posterior distributions including fractional-order parameters for dielectric elastomers, demonstrating the feasibility and value of this approach for fractional-order models of soft materials.

## Results and Discussion

Our Bayesian analysis yielded well-converged posterior distributions for all model parameters. It provided excellent fits to the experimental impedance data and produced credible uncertainty bounds that successfully encompass independent validation measurements.

### Prior Distribution Construction

The construction of appropriate prior distributions is a critical step in Bayesian analysis. Our priors were designed to be informative but not overly restrictive. This approach allows the data to substantially influence the posterior while incorporating reasonable physical constraints and supporting the knowledge gained from the deterministic optimization.

For the fractional-order exponents ($\alpha _m$, $\alpha _{sc}$, $\alpha _{imj}$, $\alpha _{tm}$), we assigned truncated normal priors with bounds between 0.5 and 0.999. These bounds ensure that the fractional derivatives remain physically meaningful. The means of these priors were set at the previously optimized values from the deterministic optimization. This reflects our confidence that these values are approximate while acknowledging uncertainty.

The mechanical and electrical parameters including stiffness coefficients ($K_{mt}$, $K_{sc}$, $K_{imj}$, $K_{mec}$), resistance values ($R_{mt}$, $R_{sc}$, $R_{imj}$, $R_{mec}$), mass parameters ($M_{mt}$, $M_{sc}$, $M_{mec}$), and characteristic impedances ($Z_{0tm}$, $Z_{0ec}$) were given normal prior distributions. The prior means were set to the deterministic optimization results, and the prior standard deviations were set to 10–20% of the absolute parameter values. We do not expect parameters to vary substantially from their optimized values, but we acknowledge that the optimization may not have found the absolute global minimum or that individual variability exists.

An important practical consideration in MCMC sampling is the numerical stability. Parameters in the model span many orders of magnitude, from very small time constants to large stiffness values. To keep all parameters within computationally stable ranges during the sampling, we applied numerical scaling factors ranging from $10^{-10}$ to $10^{6}$. For instance, the geometric length parameter $l_{ec}$ was scaled by $10^3$, and certain time constants were scaled by $10^6$. These scalings are purely computational conveniences and do not affect the physical interpretation of results—we simply reverse the scaling when reporting final parameter estimates.

The observation noise parameters ($\sigma _{real}$, $\sigma _{imag}$) governing the measurement uncertainty in the real and imaginary components of impedance were assigned half-normal priors with scales set to approximately 3% of the respective impedance component standard deviations. This reflects the expected measurement precision of the Mimosa Acoustics reflectance system, which has been characterized in previous studies [48].

### Parameter Estimation

Figure 3 shows the posterior distributions for the four fractional-order exponents in the model for subject NDP11. Each panel shows the posterior probability density for one of the fractional-order parameters: (a) $\alpha _m$ governing the malleus-incus complex, (b) $\alpha _{sc}$ for the stapes-cochlea connection, (c) $\alpha _{imj}$ for the incudostapedial and incudomalleolar joints, and (d) $\alpha _{tm}$ for the tympanic membrane. The vertical dashed line indicates the posterior mean, and the shaded region represents the 95% credible interval.

The posterior distributions exhibit tight, unimodal shapes, indicating that the experimental data are highly informative for these parameters. The unimodal and approximately Gaussian nature of these posterior distributions suggests that simpler approaches, such as using the curvature of the log-likelihood (Fisher information), could provide reasonable estimates of parameter uncertainty in future applications. For this subject, we find $\alpha _m = 0.840 \pm 0.009$, characterizing the viscoelastic behavior of tissues associated with the malleus. This value indicates substantial viscoelastic effects consistent with the presence of ligaments and muscles attached to this ossicle. The parameter governing the viscoelasticity of the stapes-cochlea connection, $\alpha _{sc} = 0.980 \pm 0.010$, is very close to one, and given the low standard deviation, this provides strong confidence in a dominant elastic component of the response, consistent with the mechanical behavior of the annular ligament supporting the stapes footplate. The exponent $\alpha _{imj} = 0.963 \pm 0.015$ characterizes the ossicular joints, ligaments, and muscles, with this near-unity value reflecting dominant elastic behavior arising from the complex joint tissues and surrounding ligaments that permit ossicular mobility while providing support and dissipating energy. Finally, $\alpha _{tm} = 0.914 \pm 0.020$ describes the tympanic membrane’s viscoelastic characteristics, which is expected given its composite structure of collagen fibers embedded in a viscoelastic matrix. Overall, the narrow credible intervals across parameters indicate strong data informativeness and robust inference.

**Fig. 4:**
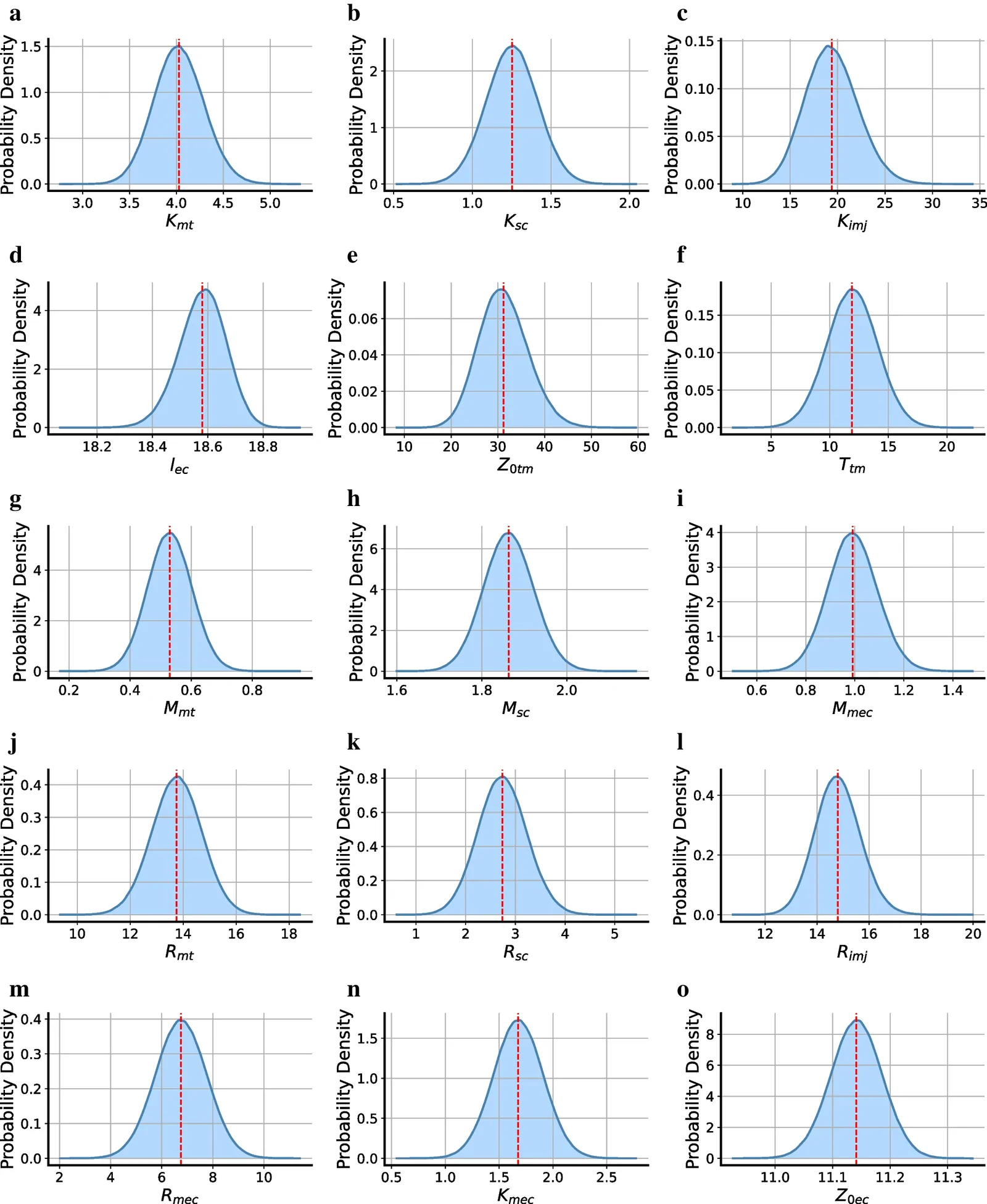
Posterior distributions for model parameters of NDP11 subject. The parameters include: stiffness coefficients ($K_{mt}$, $K_{sc}$, $K_{imj}$, $K_{mec}$), the ear canal length ($l_{ec}$), characteristic impedances ($Z_{0tm}$, $Z_{0ec}$), the tympanic membrane time constant ($T_{tm}$), mass parameters ($M_{mt}$, $M_{sc}$, $M_{mec}$), and resistance values ($R_{mt}$, $R_{sc}$, $R_{imj}$, $R_{mec}$)

Figure 4a–o presents the posterior distributions for the remaining model parameters for subject NDP11, including mechanical stiffnesses, resistances, masses, characteristic impedances, and geometric dimensions. Each panel shows the histogram, posterior mean, and 95% credible interval.

All posterior distributions exhibit well-defined, unimodal shapes with reasonable uncertainty bounds. The posterior distributions for the stiffness coefficients show a moderate spread, with coefficients of variation typically in the range of 10–25%. For the NDP11 subject, we observe $K_{mt} = 4.029 \pm 0.266$, $K_{sc} = 1.252 \pm 0.163$, and $K_{imj} = 19.378 \pm 2.762$, which are consistent with the expected variability in biological systems and the uncertainty of measurements. The geometric parameter $l_{ec} = 18.58 \pm 0.085$ mm shows a very tight posterior distribution with less than 0.5% uncertainty. This makes sense as the length of the ear canal is a relatively stable anatomical dimension that strongly influences the acoustic resonances observed in the impedance measurements. The mass and impedance parameters show posteriors with coefficients of variation ranging from 10 to 25%, similar to the stiffness parameters. The characteristic impedances $Z_{0tm}$ and $Z_{0ec}$ are particularly important because they influence the impedance transformations at the key interfaces in the system. The symmetric, bell-shaped nature of all these posterior distributions, without significant skewness or multimodality, confirms that the MCMC sampling has adequately converged and thoroughly explored the posterior distribution. This successful parameter estimation provides confidence that the Bayesian framework has appropriately quantified both the central tendency and the uncertainty for all model parameters. Table 1 summarizes the posterior parameter estimates for all seven subjects in the study. Values are scaled, as indicated in the first column, to maintain numerical precision during the computation. This table provides a comprehensive view of inter-subject variability and allows comparison of parameter estimates across individuals.

Several important observations can be made from this cross-subject comparison. There is substantial inter-subject variability in parameter values. This is expected given the natural anatomical and physiological differences between individuals, where stiffness parameters vary by factors of 2–5 across subjects, and fractional-order exponents show ranges from approximately 0.5 to 1.0 depending on the specific parameter and subject. Despite this inter-subject variability in parameter means, the within-subject uncertainties remain relatively consistent, typically 5–25% of the parameter values. This suggests that the Bayesian estimation procedure reliably quantifies uncertainty regardless of the specific parameter values. The length of the ear canal shows remarkably consistent values in most subjects ranging from approximately 18.6 to 22.6 mm, with subject NDP12 as an outlier at 8.9 mm with very tight uncertainty bounds. DP12 was female, and the slightly smaller ear canal size may be associated with individual anatomical differences. Although fractional exponents vary between subjects, there are interesting patterns, where $\alpha _{sc}$ tends to be higher than $\alpha _{imj}$ in most subjects, reflecting differences in their viscoelastic properties. This comprehensive table provides not only a valuable reference for future modeling efforts but also demonstrates the success of the Bayesian approach in handling a diverse population of subjects with varying auditory anatomical and biomechanical properties.

### Impedance Fitting Performance

**Fig. 5:**
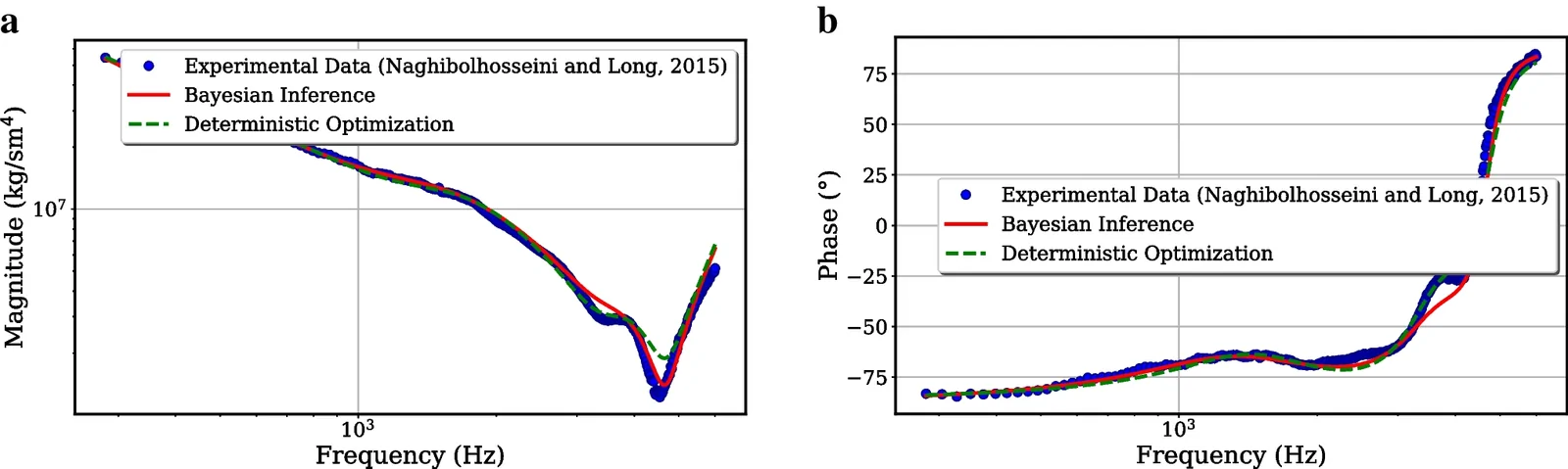
Impedance fitting for Subject NDP1: (**a**) impedance magnitude and (**b**) impedance phase, showing the Bayesian inference (solid red line), deterministic optimization (dashed green line), and experimental data (blue dots) by [40]

**Fig. 6:**
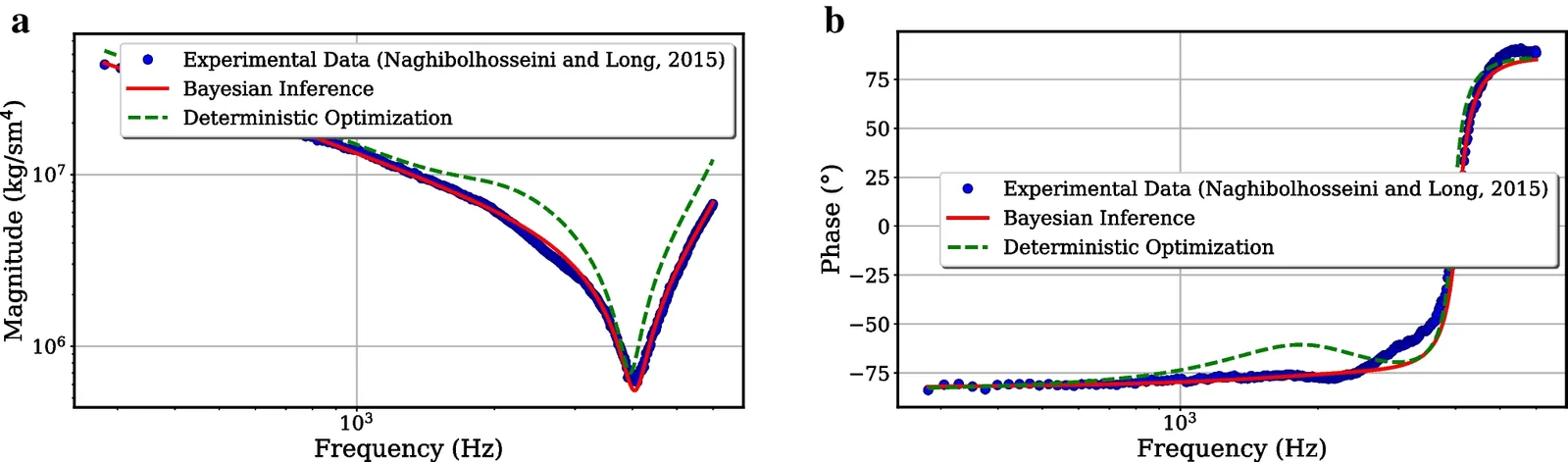
Impedance fitting for Subject NDP2: (**a**) impedance magnitude and (**b**) impedance phase, showing the Bayesian inference (solid red line), deterministic optimization (dashed green line), and experimental data (blue dots) by [40]

**Fig. 7:**
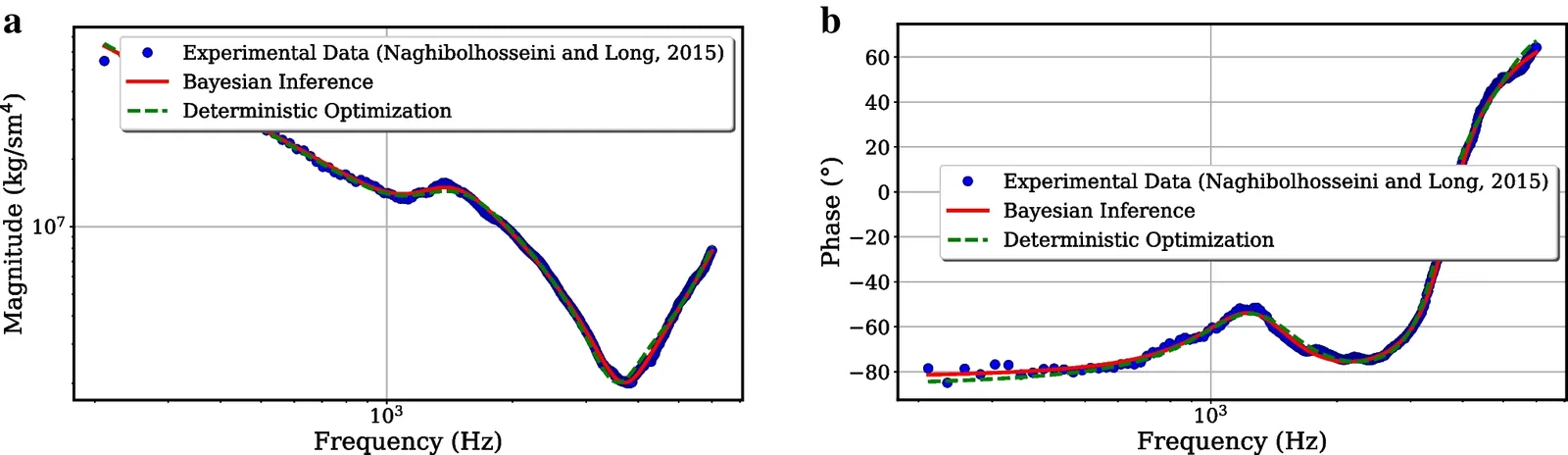
Impedance fitting for Subject NDP3: (**a**) impedance magnitude and (**b**) impedance phase, showing the Bayesian inference (solid red line), deterministic optimization (dashed green line), and experimental data (blue dots) by [40]

**Fig. 8:**
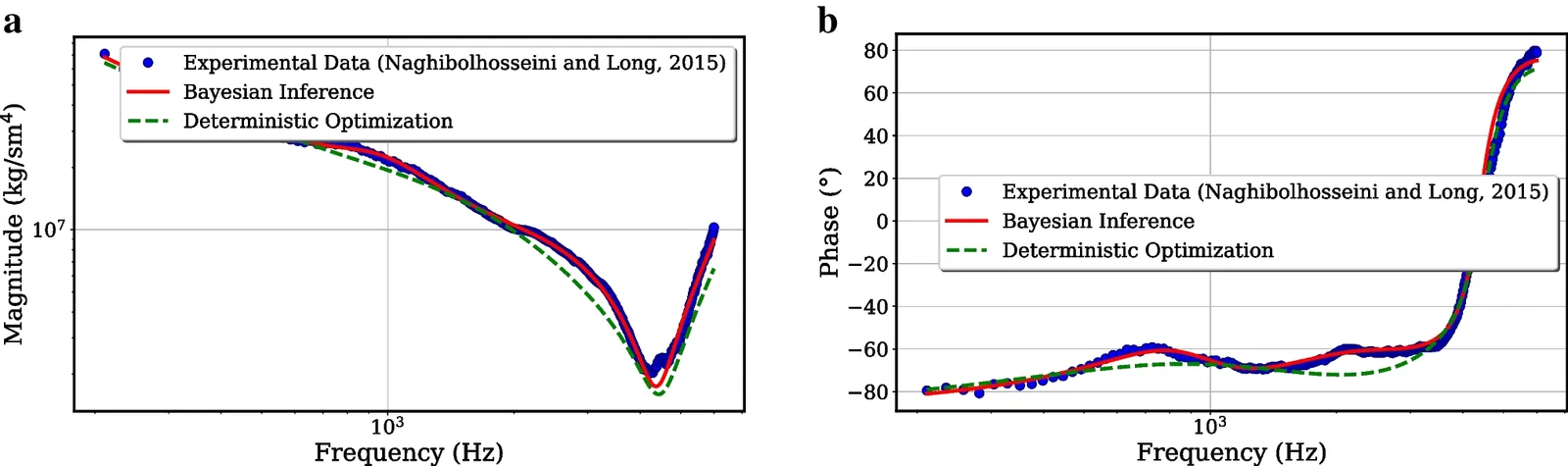
Impedance fitting for Subject NDP5: (**a**) impedance magnitude and (**b**) impedance phase, showing the Bayesian inference (solid red line), deterministic optimization (dashed green line), and experimental data (blue dots) by [40]

**Fig. 9:**
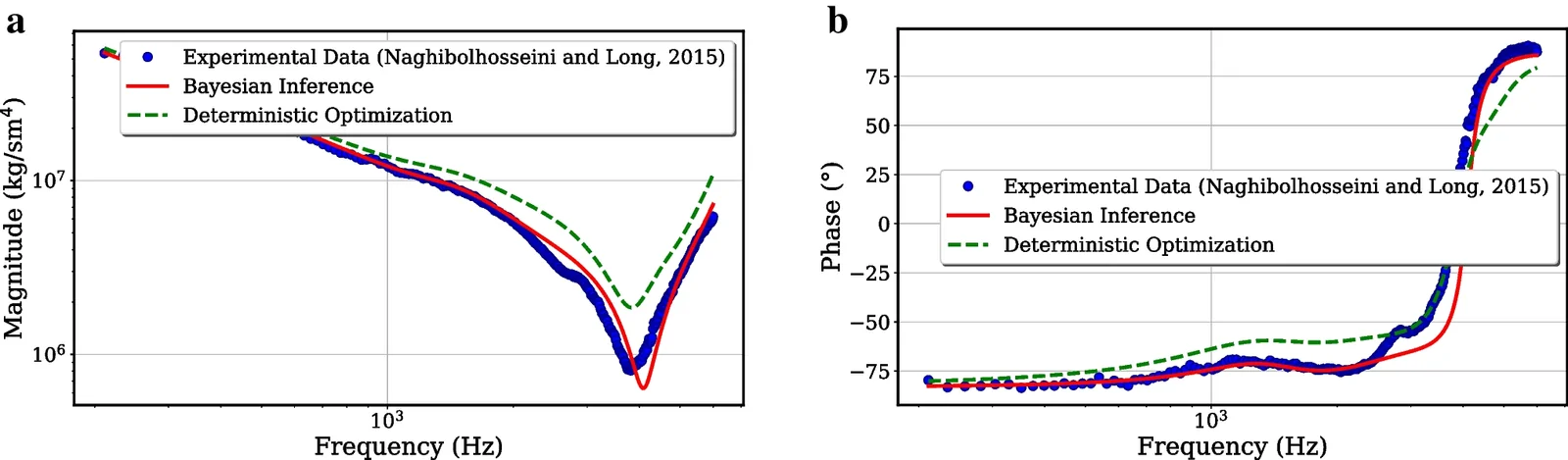
Impedance fitting for Subject NDP6: (**a**) impedance magnitude and (**b**) impedance phase, showing the Bayesian inference (solid red line), deterministic optimization (dashed green line), and experimental data (blue dots) by [40]

**Fig. 10:**
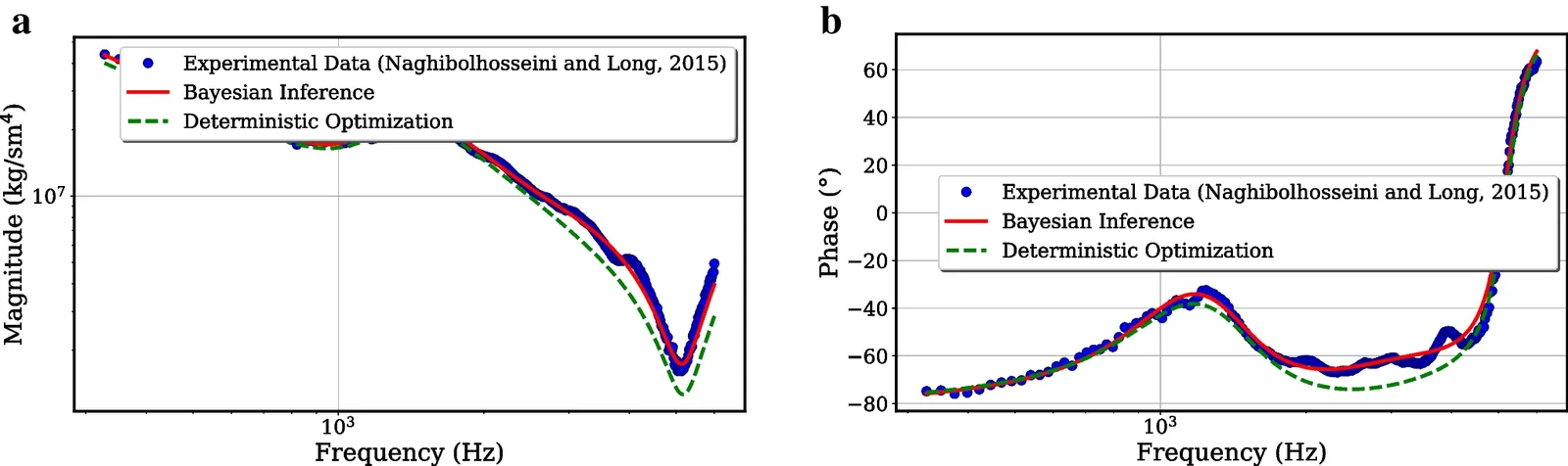
Impedance fitting for Subject NDP11: (**a**) impedance magnitude and (**b**) impedance phase, showing the Bayesian inference (solid red line), deterministic optimization (dashed green line), and experimental data (blue dots) by [40]

**Fig. 11:**
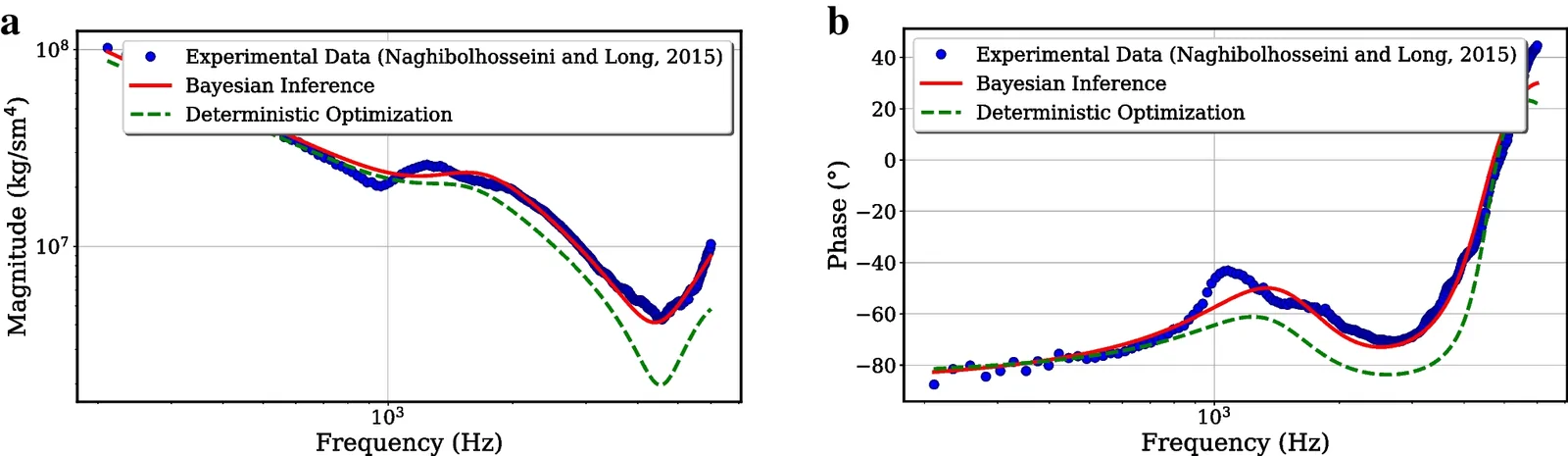
Impedance fitting for Subject NDP12: (**a**) impedance magnitude and (**b**) impedance phase, showing the Bayesian inference (solid red line), deterministic optimization (dashed green line), and experimental data (blue dots) by [40]

Figures 5, 6, 7, 8, 9, 10, and 11 show the experimental impedance measurements used for the parameter estimation, along with the impedance magnitude and phase predictions by the Bayesian inference using posterior mean parameter values in comparison with the previous deterministic optimization results for all seven subjects. For each subject, the left panel (a) shows the impedance magnitude versus frequency on a log-log plot, while the right panel (b) displays the impedance phase versus frequency on a semi-log plot. The experimental data are overlaid with the Bayesian inference predictions and the deterministic optimization (as seen in the legends).

**Table 1:** Posterior parameter estimates (mean ± SD) with scaled values for all subjects

Parameter	NDP1	NDP2	NDP3	NDP5	NDP6	NDP11	NDP12
$K_{mt}$ ($\times 10^{10}$)	14.623±0.94	5.654±0.98	4.857±0.303	6.994±0.5	2.759±0.19	4.029±0.266	2.912±0.131
$K_{sc}$ ($\times 10^{10}$)	4.879±0.893	2.669±0.402	0.943±0.15	1.792±0.2	6.226±0.731	1.252±0.163	1.14±0.054
$K_{imj}$ ($\times 10^{10}$)	18.288±2.081	14.655±1.922	15.984±2.057	8.51±0.665	0.915±0.059	19.378±2.762	51.524±2.375
$K_{mec}$ ($\times 10^{9}$)	8.389±1.595	6.628±1.019	9.028±1.492	8.829±0.554	26.698±4.548	1.678±0.231	111.123±3.717
$l_{ec}$ ($\times 10^{-3}$)	21.213±0.05	22.245±0.033	21.656±0.117	20.747±0.092	22.507±0.086	18.58±0.085	8.919±0.627
$Z_{0tm}$ ($\times 10^{6}$)	135.482±17.721	123.359±23.642	64.78±7.661	31.635±1.869	4.145±0.43	31.216±5.258	3.227±0.318
$Z_{0ec}$ ($\times 10^{6}$)	9.156±0.029	6.61±0.021	9.099±0.046	10.898±0.04	6.644±0.031	11.141±0.045	11.73±0.188
$T_{tm}$ ($\times 10^{-6}$)	13.461±1.928	13.484±2.937	16.054±3.398	29.083±2.471	2.469±0.63	11.901±2.161	10.153±0.495
$M_{mt}$ ($\times 10^{3}$)	0.708±0.121	0.679±0.103	0.201±0.03	0.667±0.06	1.305±0.116	0.53±0.072	0.206±0.01
$M_{st}$ ($\times 10^{3}$)	2.234±0.103	1.243±0.133	2.562±0.078	2.584±0.08	2.264±0.136	1.863±0.059	2.29±0.078
$M_{mec}$ ($\times 10^{3}$)	0.287±0.055	0.305±0.047	0.194±0.03	0.436±0.049	1.085±0.141	0.992±0.1	0.169±0.008
$R_{mt}$ ($\times 10^{6}$)	4.984±1.096	7.108±0.811	9.784±1.221	16.015±1.168	3.12±0.329	13.753±0.937	5.783±0.337
$R_{sc}$ ($\times 10^{6}$)	25.463±1.421	19.366±1.521	3.58±0.719	5.44±0.628	5.117±0.841	2.739±0.492	11.065±0.454
$R_{imj}$ ($\times 10^{6}$)	0.946±0.197	0.85±0.128	12.107±0.844	5.267±0.364	0.965±0.1	14.796±0.866	0.01±0.001
$R_{mec}$ ($\times 10^{6}$)	5.31±1.143	11.232±1.107	0.983±0.15	18.42±1.212	15.087±1.297	6.755±1.005	6.747±0.439
$\alpha _{m}$	0.949±0.007	0.991±0.003	0.813±0.008	0.961±0.005	0.738±0.009	0.84±0.009	0.907±0.01
$\alpha _{sc}$	0.992±0.01	0.705±0.016	0.99±0.01	0.912±0.005	0.985±0.009	0.98±0.01	0.859±0.013
$\alpha _{imj}$	0.887±0.012	0.538±0.03	0.895±0.014	0.956±0.005	0.669±0.009	0.963±0.015	0.909±0.008
$\alpha _{tm}$	0.91±0.018	0.59±0.029	0.896±0.02	0.958±0.005	0.875±0.022	0.914±0.02	0.995±0.001

Across all seven subjects, the Bayesian inference accurately captures both the magnitude and phase characteristics of the measured ear impedance across the entire frequency range from approximately 200 Hz to 6 kHz. Table 2 presents a comprehensive comparison of the fitting performance between the Bayesian inference and deterministic optimization across all subjects, quantifying the accuracy using normalized root-mean-square error (NRMSE) for both impedance magnitude and phase.

**Table 2:** Comparison of impedance fitting performance between Bayesian inference and deterministic optimization

	Magnitude NRMSE (%)		Phase NRMSE (%)	
Subject	Bayesian	Deterministic	Bayesian	Deterministic
NDP1	3.33	4.00	9.00	9.00
NDP2	2.59	19.00	7.84	16.00
NDP3	5.69	6.00	2.73	5.00
NDP5	2.67	9.00	8.54	9.00
NDP6	5.31	19.00	18.25	19.00
NDP11	3.45	9.00	6.45	11.00
NDP12	6.22	12.00	10.12	21.00

The results show substantial improvement of the Bayesian inference over the deterministic optimization. Subjects NDP2, NDP6, and NDP12 show particularly significant improvements in magnitude fitting, where the Bayesian inference reduces errors by factors ranging from 1.9 to 7.3. The magnitude error drops from 19.00% to 2.59%, and the phase error decreases from 16.00% to 7.84% for NDP2. Even in cases where both methods perform well, such as NDP1 and NDP3, the Bayesian inference maintains better accuracy. This consistent improvement across subjects reflects several key advantages of the Bayesian framework: the fractional-order elements more accurately capture the viscoelastic behavior of biological tissues, the HMC sampling procedure efficiently explores the parameter space to identify optimal parameter combinations, and the probabilistic framework naturally incorporates the parameter uncertainty.

### Outer-Middle Ear Transfer Function and Gain

In this section, we examine the model’s predictions for the forward and reverse outer-middle ear transfer functions and total gain. In this study, the outer ear only included the ear canal. These quantities characterize how efficiently sound is transmitted through the ear canal and middle ear to reach the cochlea (forward direction) and how effectively cochlear emissions propagate back out to the ear canal (reverse direction).

**Fig. 12:**
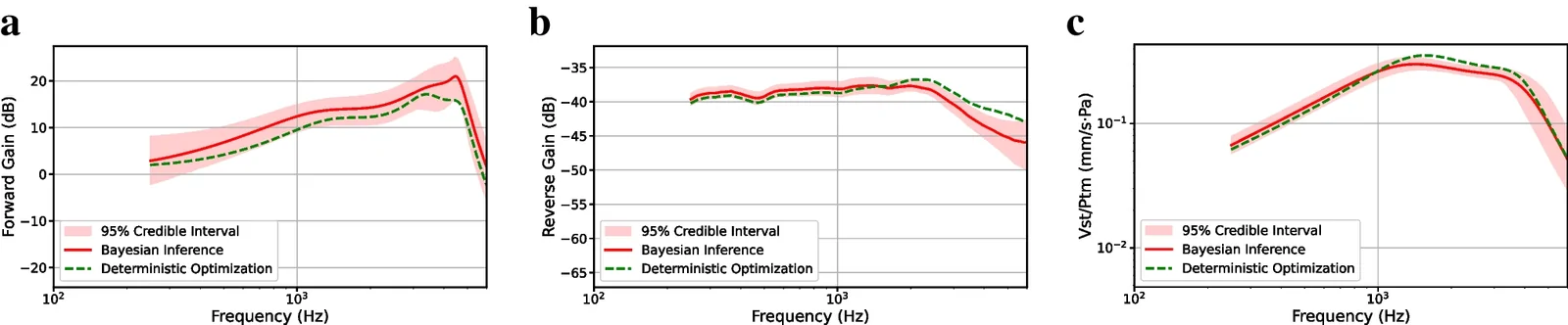
Forward (**a**), reverse (**b**), and stapes velocity transfer function (**c**) for NDP1

Figures 12 through 18 present comprehensive transfer function analyses for all seven subjects. Each figure contains three panels showing (a) the forward transfer function magnitude representing sound transmission from the ear canal to the cochlea, (b) the reverse transfer function magnitude showing transmission from the cochlea back to the ear canal, and (c) the stapes velocity transfer function characterizing the velocity of the stapes footplate relative to the ear canal pressure. The shaded regions in each panel represent 95% credible intervals from the Bayesian posterior distribution, illustrating how parameter uncertainty propagates to prediction uncertainty.

**Fig. 13:**
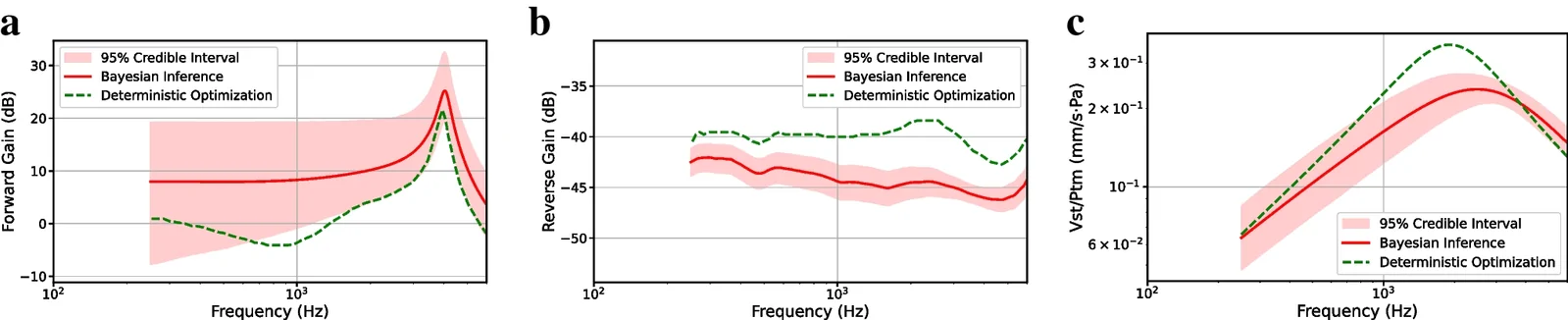
Forward (**a**), reverse (**b**), and stapes velocity transfer function (**c**) for NDP2

**Fig. 14:**
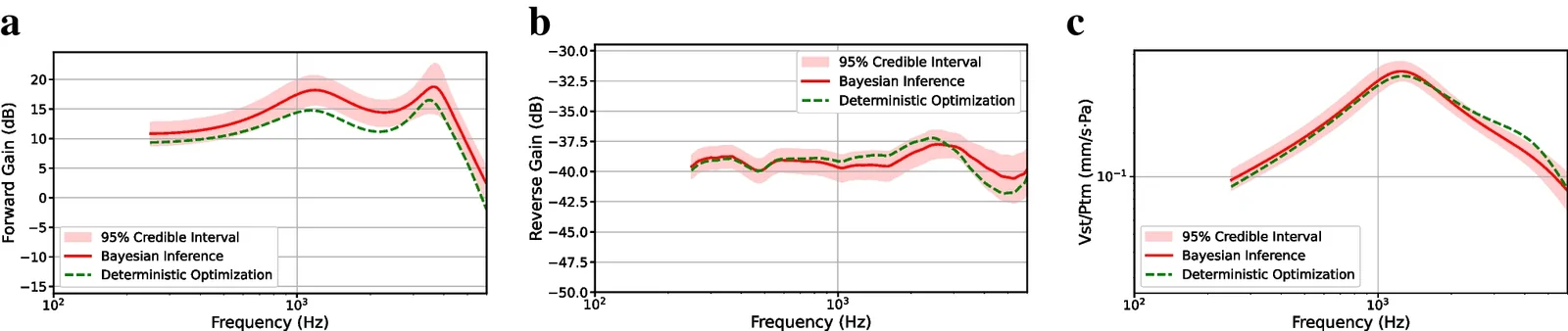
Forward (**a**), reverse (**b**), and stapes velocity transfer function (**c**) for NDP3

**Fig. 15:**
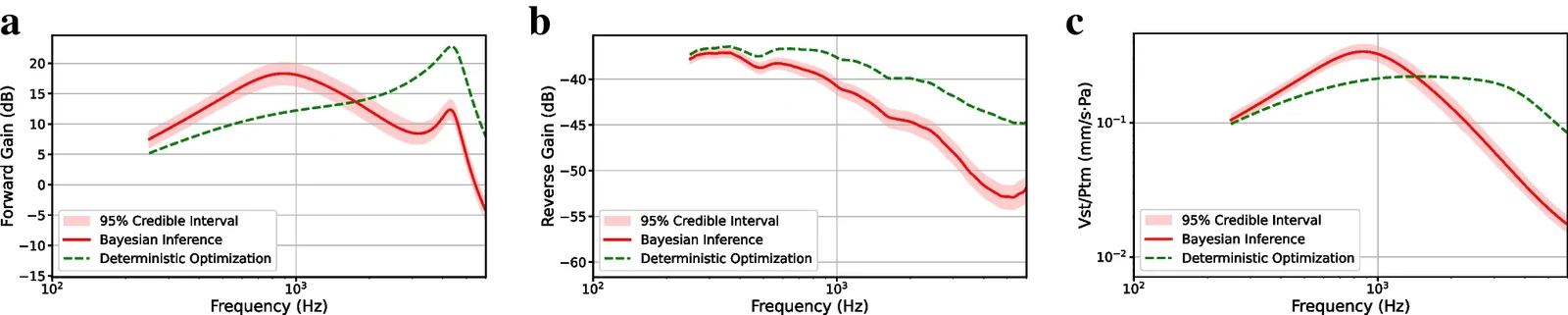
Forward (**a**), reverse (**b**), and stapes velocity transfer function (**c**) for NDP5

**Fig. 16:**
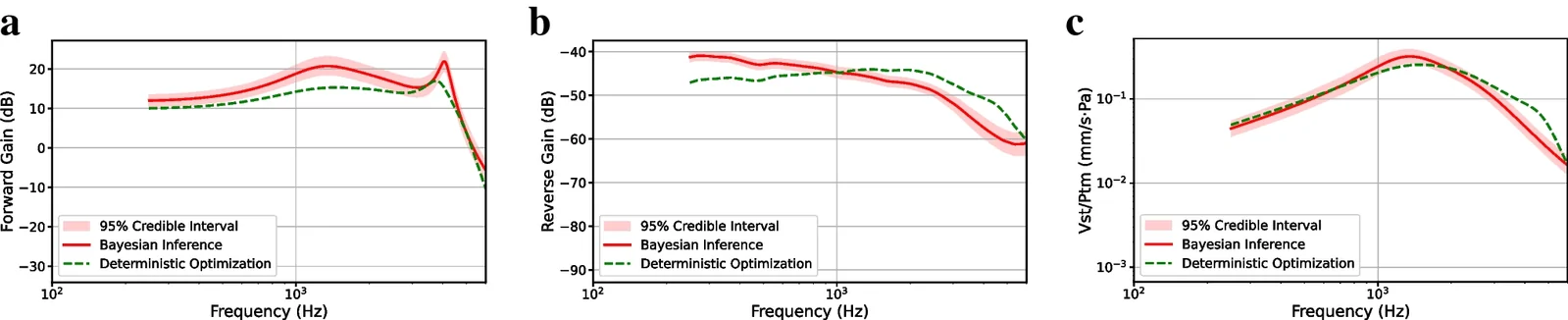
Forward (**a**), reverse (**b**), and stapes velocity transfer function (**c**) for NDP6

**Fig. 17:**
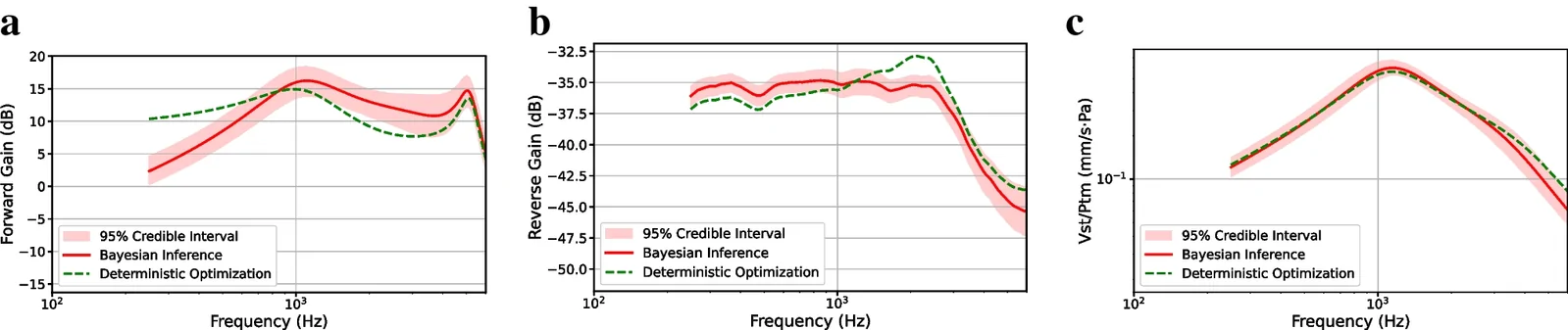
Forward (**a**), reverse (**b**), and stapes velocity transfer function (**c**) for NDP11

**Fig. 18:**
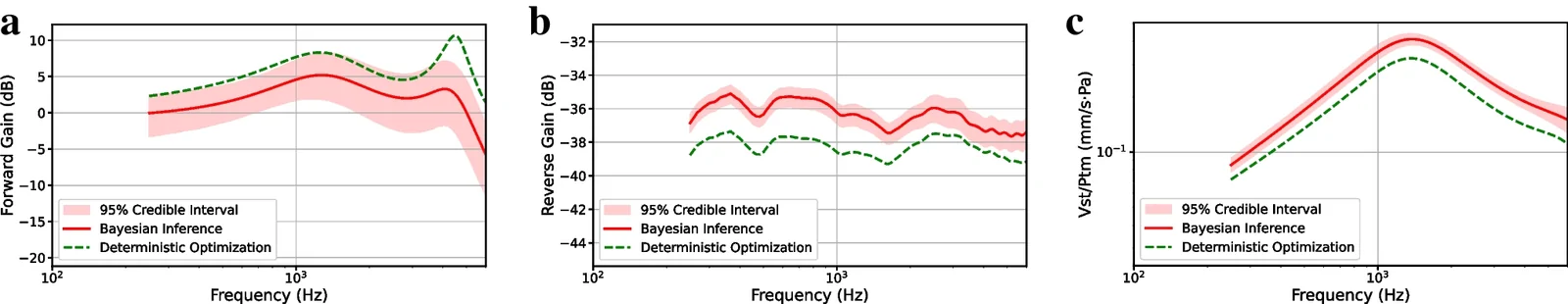
Forward (**a**), reverse (**b**), and stapes velocity transfer function (**c**) for NDP12

As can be seen in Figs. 12, 13, 14, 15, 16, 17, and 18, for several subjects and transfer functions, the deterministic optimization estimates fall within the Bayesian 95% credible intervals, indicating agreement between the two approaches and suggesting that the deterministic solutions are consistent with the posterior uncertainty captured by the Bayesian inference. In other cases, the deterministic predictions lie partially or entirely outside the credible intervals, reflecting discrepancies that may arise from point-estimate assumptions in the deterministic optimization and its lack of explicit uncertainty quantification. Overall, these differences highlight the added value of the Bayesian inference in characterizing parameter uncertainty and capturing intersubject variability, particularly in cases where deterministic estimates do not fully align with the probabilistic model predictions.

**Fig. 19:**
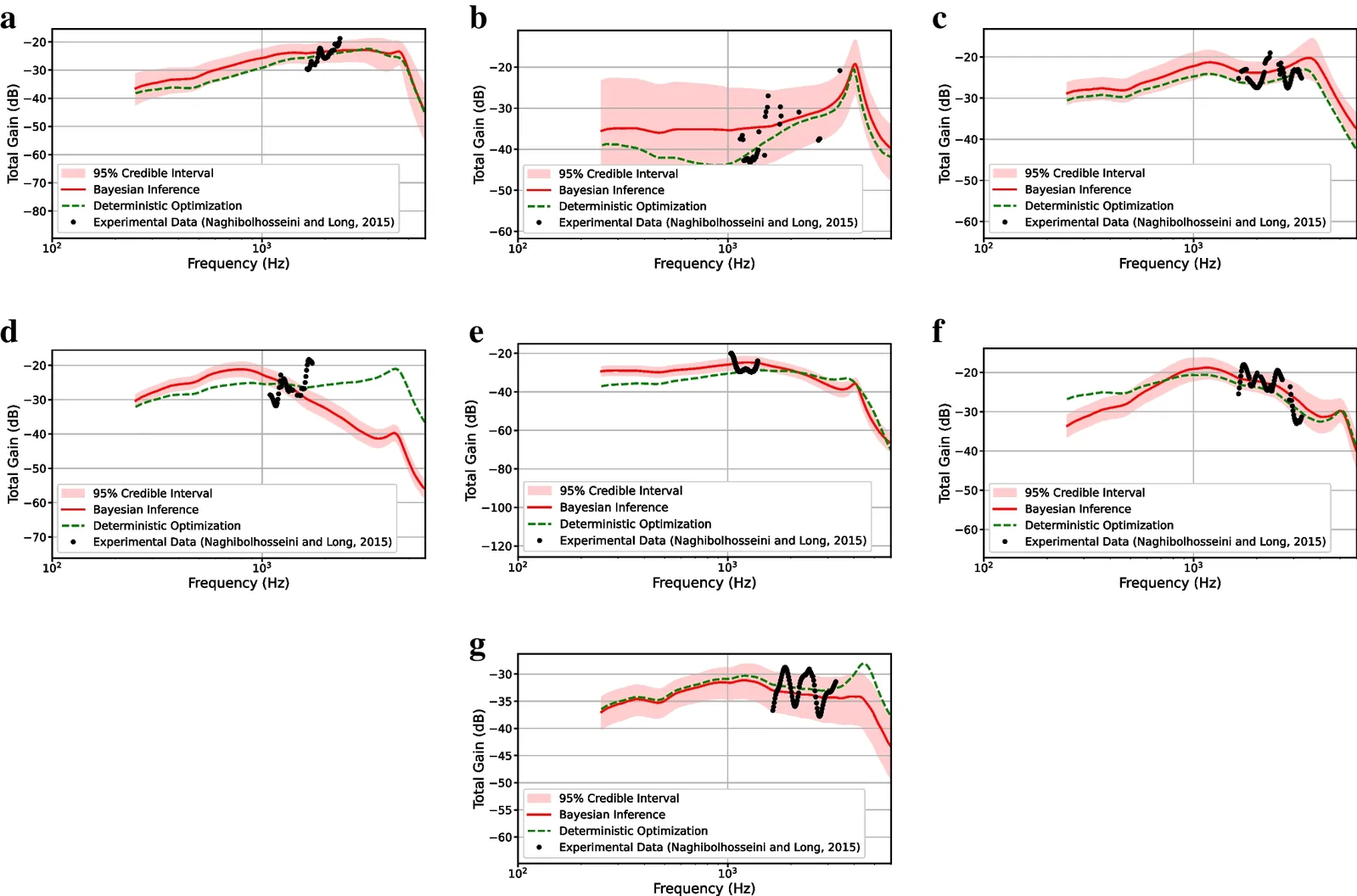
Model validation against experimental data for all seven subjects: (**a**) NDP1, (**b**) NDP2, (**c**) NDP3, (**d**) NDP5, (**e**) NDP6, (**f**) NDP11, and (**g**) NDP12. The graphs show the Bayesian inference OMEG estimates (red solid lines), 95% credible intervals (shaded red regions), the estimates by the deterministic optimization (dashed green lines), and the experimental data (black dots) by [40]

Figure 19a–g shows comparisons between the predicted total outer-middle-ear gain (OMEG) of the model and experimental OMEG estimates derived from DPOAE measurements for all seven subjects. In each panel (a–g), the experimental DPOAE-based OMEG estimates are shown by black dots, the predicted OMEG by the Bayesian inference is shown as a solid red line with a shaded 95% credible interval, and the deterministic optimization predictions are shown by dashed green lines.

This validation is important because the OMEG experimental measurements come from a different and independent method than the reflectance measurements, used to estimate the model parameters. The OMEG estimation requires accurate modeling of both forward and reverse sound transmission rather than just the input impedance, and any errors in the model structure or parameter estimates will accumulate through the round-trip path. The results demonstrate excellent agreements across all subjects and frequencies. The model’s 95% credible intervals fully encompass the vast majority of experimental OMEG data points across the entire frequency range. This indicates that the Bayesian uncertainty quantification is well-calibrated with credible intervals that are neither too narrow nor too wide. The agreement between the Bayesian inference and experiment is maintained between approximately 1 and 3.3 kHz, where the OMEG experimental data is available. The predicted OMEG values range between –39 and –17 dB, consistent with experimental observations. This frequency range is also important for hearing, as it includes the region of highest auditory sensitivity. The model accurately captures subject-specific OMEG behavior: for example, NDP1 exhibits a relatively high and stable gain (around –20 dB), whereas NDP11 shows a more frequency-dependent response with a peak near 2 kHz. These differences arise naturally from the subject-specific parameters estimated through Bayesian inference.

The Bayesian inference predictions closely match the deterministic optimization results, and the credible intervals successfully encompass nearly all experimental data points. This excellent agreement across all subjects confirms that the model reliably predicts independent experimental OMEG measurements that were not used during the parameter estimation, demonstrating both a strong predictive capability and well-calibrated uncertainty quantification.

### Model Comparison

In this section, we compare stapes velocity transfer function and ear canal gain with existing literature.

**Fig. 20:**
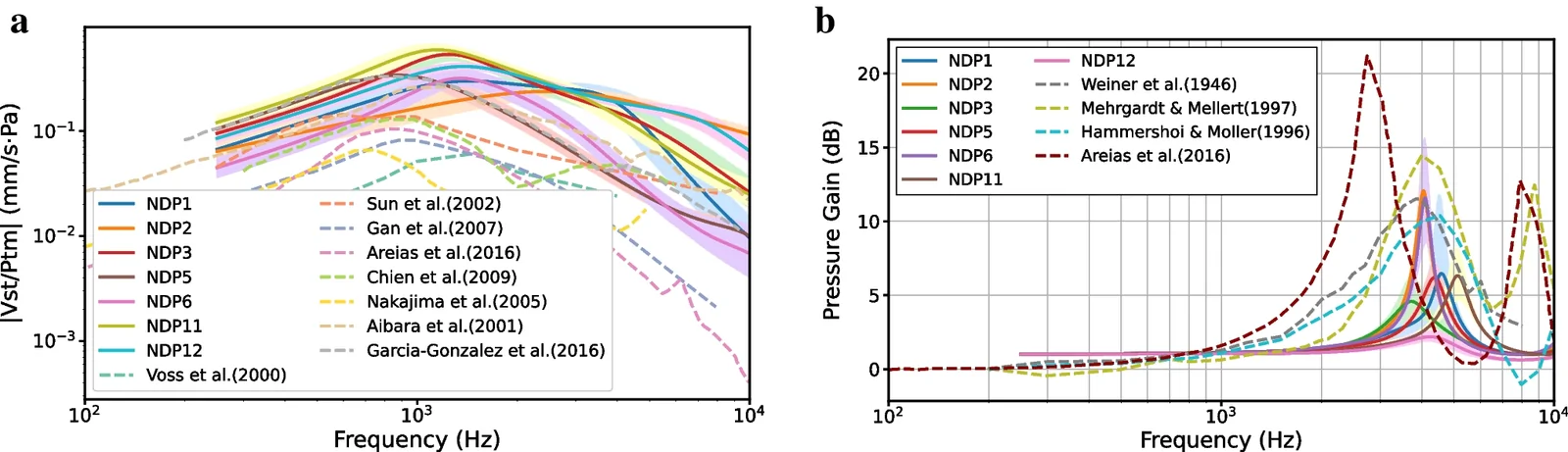
Model validation against experimental and computational data. (**a**) Stapes velocity transfer function [49–56] and (**b**) ear canal pressure gain [55, 57–59] for seven subjects compared with literature as listed in the legend

Figure 20a shows that the present model exhibits a primary resonance near 1 kHz and a secondary elevation around 3–5 kHz. This prediction is consistent with experimental measurements by Voss et al. [49], obtained from 18 normal hearing adult human cadaveric ears, Aibara et al. [50], Nakajima et al. [51], via intracochlear pressure recordings, and Chien et al. [52], based on human and mammalian middle ear transfer function data. Earlier computational predictions by Sun et al. [53] and Gan et al. [54] reproduced the general low-frequency behaviors, but underestimated the secondary resonance, which more recent cavity-inclusive finite-element models by Areias et al. [55] and Garcia Gonzalez et al. [56] captured more clearly. The Bayesian predictions exhibit higher stapes velocity per tympanic membrane pressure above 7 kHz, a frequency region where larger intersubject variability is expected due to anatomical and viscoelastic differences. The variability across parameter sets falls within the experimentally reported intersubject spread, indicating that the Bayesian framework captures realistic biological variations.

Figure 20b presents the ear canal pressure gain, which shows a primary resonance between 2.5 and 4 kHz with peak gains of 4–12 dB. These values are lower than the classical results of Wiener et al. [57] (average eardrum pressure ratio in adult males) and Hammershøi and Møller [58] (DPOAE-derived gain, with peaks above 20 dB near 3 kHz), but our predictions align more closely with Mehrgardt and Mellert [59], who measured a resonance near 5 kHz with gains around 14 dB using an impulse-response–based measurement approach. The present model does not reproduce the higher frequency secondary resonance at approximately 8 kHz, observed in the finite-element simulations of Areias et al. [55] or at around 9 kHz, produced in the experimental study by Mehrgardt and Mellert [59]. However, it should be noted that this second resonance was not also observed by Hammershøi and Møller [58]. Moreover, it should be noted that parameter estimation for the current model was performed using impedance data up to 6 kHz. Extending the experimental dataset to a broader frequency range would enable further evaluation and support of the model’s performance at higher frequencies. Comparison with a detailed finite-element model based on the reconstruction of the tympanic membrane, ossicular chain, cochlear load, tendons, ligaments, middle ear joints, portions of the temporal bone, the external auditory meatus, the tympanic cavity, and surrounding soft tissues, the fractional-order model predictions yield lower peak gains but very low intersubject variability [55]. Together, these results demonstrate that the fractional-order Bayesian framework provides stable, physiologically consistent estimates of the ear canal gain while capturing the dominant resonance patterns observed in prior acoustic and computational studies.

**Fig. 21:**
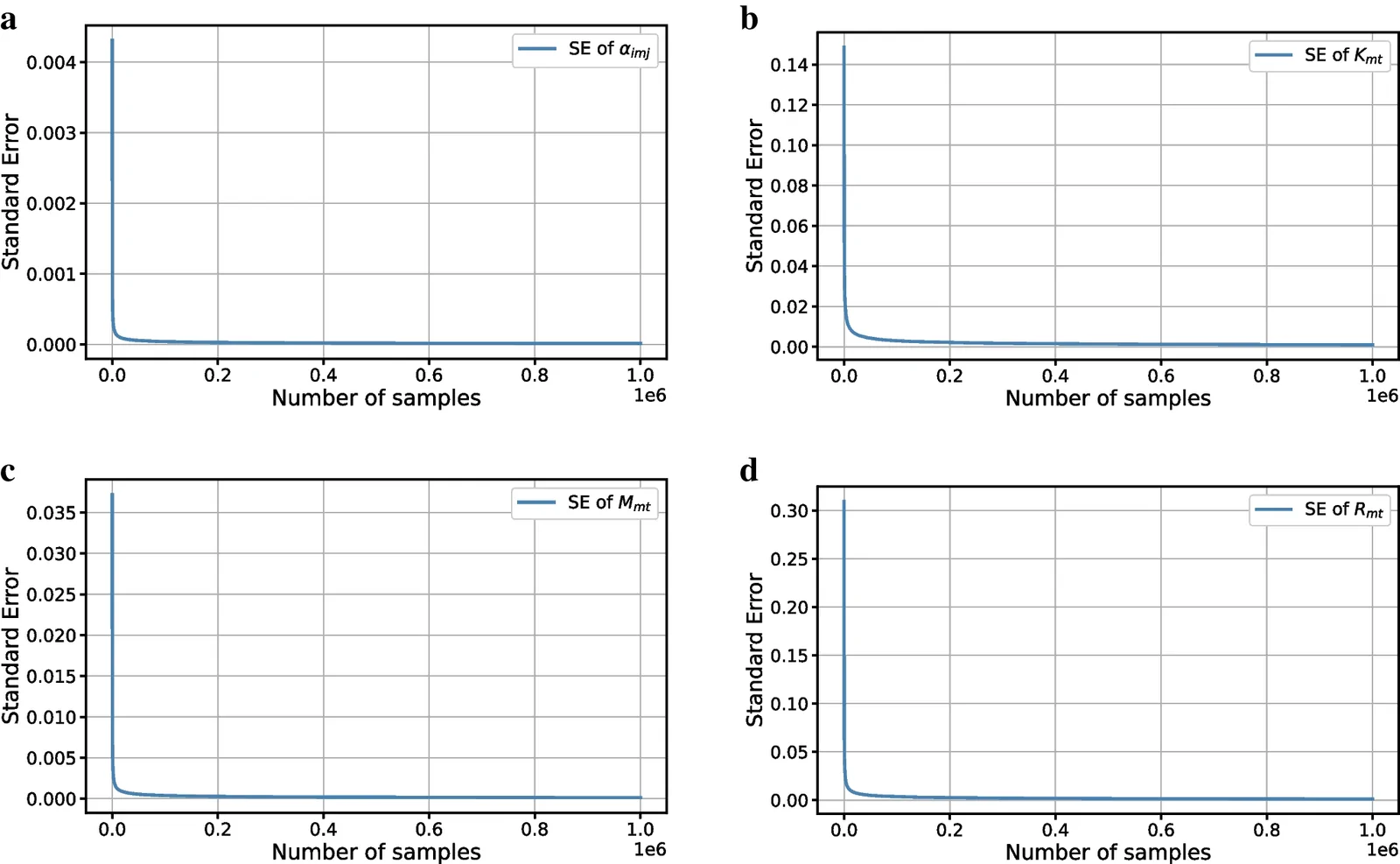
MCMC convergence diagnostics for four representative parameters of (**a**) $\alpha _{imj}$, (**b**) $K_{mt}$, (**c**) $M_{mt}$, and (**d**) $R_{mt}$

### MCMC Convergence Diagnostics

Reliable Bayesian inference requires that the MCMC sampling has adequately converged to the posterior distribution. We assessed convergence using standard diagnostics recommended by PyMC, and all 19 model parameters satisfy these criteria, indicating reliable posterior inferences.

The Gelman-Rubin statistic ($\hat{R}$) compares the variance between chains to the variance within chains, and values close to 1.0 indicate that all chains have converged to the same distribution. All parameters achieve $\hat{R} \le 1.01$, which is well below the commonly used threshold of 1.1 and shows excellent agreement between chains. The effective sample size (ESS) adjusts the number of samples to account for the autocorrelation in the chains and reflects how many independent samples are effectively obtained. We report both bulk ESS for the central part of the distribution and tail ESS for the extreme values. All parameters achieve ESS greater than 400, which confirms adequate exploration of the posterior, including the tails where credible intervals are computed.

Hamiltonian Monte Carlo can encounter numerical issues called divergences when the posterior contains regions with very high curvature. Divergences indicate that some parts of the posterior may not be sampled correctly. We observed zero divergences across all chains, which confirms stable sampling behavior throughout the parameter space. The energy diagnostic compares the distribution of energy during sampling with the distribution during momentum resampling. All chains pass this diagnostic, providing further evidence of proper sampling.

Figure 21a–d shows the standard error of selected parameters as a function of the number of samples. Although convergence plots were produced for all 19 parameters, we present four representative cases for illustration. The standard errors decrease rapidly during the initial part of sampling and then stabilize, which indicates that the initial transient behavior has been removed and the chains are sampling from the stationary distribution. The final standard errors are small relative to the parameter values, and the smooth decrease without irregular fluctuations reflects the stable numerical performance of the Hamiltonian Monte Carlo algorithm.

Together, these diagnostics provide strong evidence that our MCMC sampling converges to the true posterior distribution. This ensures that all subsequent inferences, including parameter estimates, credible intervals, and predictive results, are statistically reliable.

## Conclusion

This study demonstrates the successful application of Bayesian inference with Hamiltonian Monte Carlo sampling to quantify parameter uncertainty in a fractional-order lumped-element model of the human ear. Building on prior deterministic modeling, we extended to a full probabilistic characterization of parameters and model predictions. All 19 model parameters exhibited well-behaved posterior distributions with tight uncertainty bounds, indicating that the experimental impedance data strongly constrain the model. Posterior mean parameter estimates improved model accuracy, and the predicted outer-middle ear gain showed excellent agreement with independent measurements, derived from DPOAE data, across subjects, with 95% credible intervals encompassing nearly all experimental data points. Convergence diagnostics confirmed reliable sampling, with $\hat{R} \le 1.01$, bulk ESS >400, and zero divergences, supporting the validity of the posterior estimates.

Comprehensive model validations against established literature further confirms the model’s physiological consistency. The Bayesian-derived parameter sets reproduce stapes velocity transfer functions with primary resonances near 1 kHz and secondary elevations around 3–5 kHz, consistent with cadaveric experimental measurements and modern cavity-inclusive finite-element models. The ear canal pressure gain predictions exhibit primary resonances between 2.5 and 4 kHz with peak gains of 4–12 dB, aligning closely with impulse response measurements while demonstrating minimal intersubject variability. The Bayesian successfully captures the dominant resonance structures across the audible frequency range. Critically, the low intersubject variability combined with realistic biological variations demonstrates that the fractional-order Bayesian framework provides stable, physiologically consistent predictions—a key requirement for the potential clinical applicability of this work in the future.

The probabilistic framework developed here provides distributions of possible outcomes rather than single predictions. This information is valuable for assessing whether a patient’s measurements fall within normal ranges, quantifying the confidence of diagnostic decisions, with potential applications in guiding surgical planning with risk assessment, personalizing hearing aid fittings based on individual ear mechanics, and monitoring disease progression or treatment response with proper statistical power.

Despite these advances, several limitations suggest directions for future research. The current study focused on subjects with normal hearing, so extending the framework to pathological conditions is an important next step. Furthermore, while seven subjects provided proof of concept, larger sample sizes would enable hierarchical Bayesian inference modeling to better characterize population-level variability and individual differences. In addition, the experimental OMEG data for the subjects included in this study were limited to the 200–6000 Hz range, corresponding to the calibrated bandwidth of the wideband reflectance measurements used in our experimental setup. Broader-band experimental measurements would enable a more comprehensive assessment of the model’s peak behavior and overall frequency-dependent trends. Incorporating additional measurements, such as the umbo velocity, intracochlear pressure, or electrocochleography, could further constrain parameters and improve predictive accuracy across broader frequency ranges.

Overall, the combination of fractional calculus, detailed biomechanical modeling, and Bayesian inference represents a powerful approach for understanding and predicting complex biological systems. This integrated framework captures the essential physics of viscoelastic biosystems, fits experimental data accurately, predicts independent validation data within calibrated uncertainty bounds across multiple acoustic measures, and provides actionable probabilistic information for decision-making. Extending this approach to pathological conditions, larger populations, and additional measurement modalities moves us closer to precision medicine in audiology, where diagnostic and therapeutic decisions are guided by personalized, validated, probabilistic models of individual patient’s auditory system physiology.

## Data Availability

Data can be provided upon request.

## Code Availability

Code can be provided upon request.
